# Dispersion Influence of Electroacoustic Transducer Parameters in the Design Process of Miniature Loudspeaker Arrays and Omnidirectional Sound Sources

**DOI:** 10.3390/s24154958

**Published:** 2024-07-31

**Authors:** Bartlomiej Chojnacki

**Affiliations:** Department of Mechanics and Vibroacoustics, AGH University of Krakow, Mickiewicza Av. 30, 30-059 Cracow, Poland; bchojnacki@agh.edu.pl

**Keywords:** drivers, loudspeaker tolerances, directivity simulations, beamforming, transducer manufacturing, sensors, quality assurance and control

## Abstract

Electroacoustic transducers represent one of the crucial materials used in the construction of loudspeaker arrays. The dispersion in their parameters may influence the performance of a speaker set. Parametric loudspeaker arrays and omnidirectional sound sources have been used for years. However, the possible influence of transducer manufacturing tolerances on the arrays’ performance has not been investigated. In previous research, the sources of possible dispersion in acoustic measurements carried out with omnidirectional sources were studied, pointing out that the problems with sound sources may be a significant reason behind the small measurement repeatability in standards. This paper investigated the measurement of several common types of miniature speakers, using 10 pieces of each type and investigating the influence of their parameter dispersion in electric and acoustic ways. Numerical simulations of omnidirectional sound sources were performed to investigate the drivers’ dispersion influence sensitivity. The results provided proof of the small-signal parameter dispersion reaching 20% of the variation. The acoustic measurements show that the loudspeakers may differ in sensitivity parameters by up to 4 dB in 10 transducer tests. The analysis of an example multitransducer array indicated that a dispersion of a sensitivity higher than 1 dB might lead to significant misperformance in constructed arrays and measurement deviations with this type of array.

## 1. Introduction

Electroacoustic drivers, like every material in engineering techniques, have tolerances and possible dispersions in technical parameters from the manufacturer’s declarations. Because of non-ideal manufacturing methods and further tolerances in basic material tolerances, such as magnets, alloys, diaphragms, or moving coils, the final loudspeaker parameters are the variables that should be considered in the multiple-transducer array design process, primarily if multiple instances of identical transducers are used in the speaker set.

**Figure 1:**
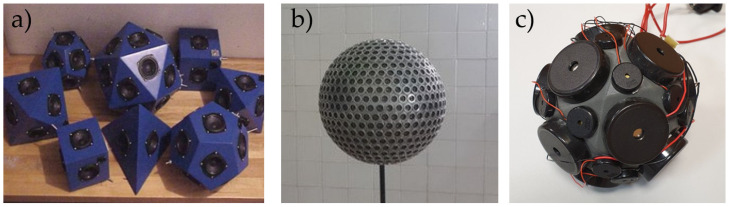
Selected samples of loudspeaker arrays: (**a**) polyhedral omnidirectional sound sources [11], (**b**) parametric loudspeaker arrays by Arnela et al. [12], (**c**) miniature omnidirectional sound source for sound insulation measurements [13].

The high-quality stereo set is the most common case in which the speakers’ dispersion in the set may affect the device performance [1]. If the sensitivity of the drivers in one set is higher than in the second one, this may lead to a significant shift in the audio scene. Also, the critical problem is the enclosure design, since, if there is a significant difference in the real and declared Thiele–Small parameters, then enclosure parameter adjustment should be considered [2]. Problems in the electroacoustic design caused by the mismatch of material parameters, such as the enclosure walls or lining, are common and well studied [3,4]. However, loudspeaker enclosures have been constructed for years, and the possible dispersions in the selected drivers have not been studied in detail. Only a few studies have been performed in this case [5], proving that the typical variation in parameters may reach 5–15%, but no comprehensive analysis has been performed. Although better transducer design and measurement methods are still being investigated [6,7], the more fundamental problems, such as tolerance for transducer manufacturing, should also be explained. The increasing popularity of advanced parametric speaker sets for research and performance purposes [8,9,10] provides a strong need for further dispersion analysis in similar constructions. Loudspeaker arrays constructed from multiple transducers may be strongly affected by possible dispersions in loudspeaker parameters. The more transducers we use, the greater the problems that may be detected [5], while some recently developed parametric speaker arrays may involve more than 100 drivers. Selected examples of loudspeaker arrays are shown in Figure 1. 

The parametric loudspeakers are investigated in wave-field synthesis methods for artificial sound field reproduction or advanced measurements in room acoustics. Previously, the impact of the sound reflections caused by speaker arrays was investigated by Zhong [14] and Zenker [15]. The review paper by Start [16] explained the difficulties in operating parametric loudspeaker arrays, where the construction troubles caused by the parameter dispersion were also elaborated. Czesak also studied the acoustic effects of multitransducer array dispersions, such as distributed-mode loudspeakers [17].

However, the loudspeaker’s directivity has been studied for years, and possible methods for its control and parametrization are still under investigation in the newest state-of-the-art papers. Despite the development of the novel array construction, DSP controlling methods are also under investigation [18,19]. The novel methods in directivity parametrization, such as spherical harmonics, allow the further development of parametric speakers [20,21]. 

One of the selected fields in parametric loudspeaker arrays comprises miniature omnidirectional sound sources. They are used in selected applications, such as near-field HRTF measurements [22,23], acoustic laboratory-scale modeling [24,25], or reduction model measurements [26]. The omnidirectional sources require very strict similarity between the transducers used in the matrix, as they are supposed to radiate the same sound wave in any direction. Then, the dispersion in electroacoustic transducers leads to significant dispersions of omnidirectional sound source performance. 

However, the topic of possible dispersions caused by deviation from the driver’s parameters seems essential to discuss; it has currently not been investigated, and its influence on loudspeaker arrays is not evident. Some research based on possible sources of dispersions in room acoustic measurements have indicated that the problems with the omnidirectional sound source driver dispersions may be significant [27,28,29], but the topic has not been studied in detail. Possible improvement in the field of transducer dispersion reduction may be achieved by increasing the development of fast measurement systems, allowing better quality control in the production line [30,31]. However, this is still difficult and not available at most factories. End users also do not know whether the transducers that they are using have been comprehensively tested and manufactured to meet low-dispersion standards. This shows the need for a detailed investigation into possible dispersions in loudspeaker parameters and their influence on loudspeaker array performance.

The current paper aims to provide information on possible levels of dispersions in measured loudspeaker parameters. By measuring 10 instances of the same loudspeaker model, parameter dispersions were investigated and marked as the variation coefficient. The influence of those dispersions was investigated in the numerical models of example loudspeaker arrays. The paper is divided into the following sections: Section 2, where the background of the transducer selection for the miniature speaker arrays is explained; Section 3, where the electric and acoustic parameters for multiple instances of the same driver are analyzed; Section 4, where, based on numerical modeling, the influence of sensitivity dispersions in omnidirectional sound sources is investigated; and Section 5, where the conclusions are reached. The paper’s outcome describes the possible problems in loudspeaker array operation caused by high loudspeaker dispersions and enables the fast assessment of the issues caused by driver parameter flow that may influence the given design.

## 2. Materials and Methods—Driver Parameters and Their Influence on the Design of Loudspeaker Arrays

Electroacoustic transducers, such as loudspeakers, are commonly described using equivalent circuit methods based on analog theory. With the development of Thiele–Small (T-S) parameters [32] and small-signal or large-signal loudspeaker parameters [6,33], it started to be possible to anticipate the performance of the actual speaker and perform advanced simulations, for example, in FEM-based software [34]. Therefore, achieving high repeatability in these parameters between the given transducer instances is essential to ensure that the possible enclosure design will function, without repeating the design process for each transducer. 

### 2.1. Electric and Acoustic Loudspeaker Parameters

The T-S parameters are typically derived from added mass or volume methods based on electrical impedance measurements [35]. The selected T-S parameters considered the most important for the current study are as follows:

- Resonant frequency—this typically means the electrical resonance frequency *f_s_*, where the electric impedance reaches the maximum value. Typically, in calculating the T-S parameters, the *f_s_′* value is also present, which is the resonant frequency for the setup with added mass or volume. With the resonant frequency parameter and maximum impedance value, the *Q_es_/Q′_es_* (electrical quality), *Qms/Q′ms* (electric quality), and *Q_ts_/Q′_ts_* (electrical quality) are derived.
- The mechanical mass of moving elements—*M_ms_* [kg]—this is calculated with Equation (1):

(1) $$M_{ms}=\frac{M}{\frac{f_{s}}{f_{s}\prime }*\frac{Q^{\prime }es}{Qes}-1},$$

where *M* is the added mass value [kg] used in the measurement procedure.

- Mechanical diaphragm compliance—*C_ms_* [s^2^/kg]—this is calculated with Equation (2):

(2) $$C_{ms}=\frac{1}{{\left(2\pi f_{s}\right)}^{2}M_{ms}}\left[\frac{s^{2}}{kg}\right].$$

Therefore, *C_ms_* may propagate more considerable errors than *M_ms_* if the *f_s_* parameter is shifted.

- Force factor—*BL* [*Tm*]—this is defined by Equation (3):

(3) $$BL=\sqrt{\frac{2\pi f_{s}R_{0}Mms}{Qes}},$$

where *R*_0_ is the driver resistance.

It can be seen that if the crucial parameters, such as *f_s_*, are shifted between the given instances of the transducers, then the error may propagate to the other parameters that are crucial to the general driver work and the enclosure design, such as VAS and mechanical resistance.

The crucial acoustic parameter considered in the current work is the sensitivity of the driver, defined as the sound power level (SPL) measured at a distance of 1 m in an anechoic environment, while the driver is powered with 1 W RMS pink noise filtered to the proper driver bandwidth [36]. This is the resulting parameter considered in the actual speaker performance. As for the loudspeaker arrays, all unit drivers must provide the same sensitivity. The study described in the current work investigates whether this assumption is achievable.

### 2.2. Loudspeaker Selection for Parametric Speaker Arrays and Omnidirectional Sound Sources

Despite the electroacoustic performance and matching sensitivity, the transducers selected for loudspeaker array development must comply with several geometrical and technical requirements, making them suitable for this type of application. It was shown that to provide a proper performance, the transducers must cover as much space as possible on the array enclosure surface [37,38]. Therefore, the following rules should be applied while the selection of the transducer for the array is carried out:

- The basket size should be limited, and the magnet should not be enormously extended in the driver’s vertical axis. Longer baskets and more significant drivers force the additional space requirements inside the enclosure to place the drivers, for example, in the sphere enclosure, and, with improper magnet and basket size, the enclosure will need to be larger and reduce the array performance.
- The effective diaphragm diameter should be as large as possible, while all additional frames, mounting elements, and other details should be limited, as they also increase the size of the driver. Moving elements should cover the highest possible space in the driver design to minimize the spatial aliasing phenomenon that is common in loudspeaker arrays [39,40].

**Figure 2:**
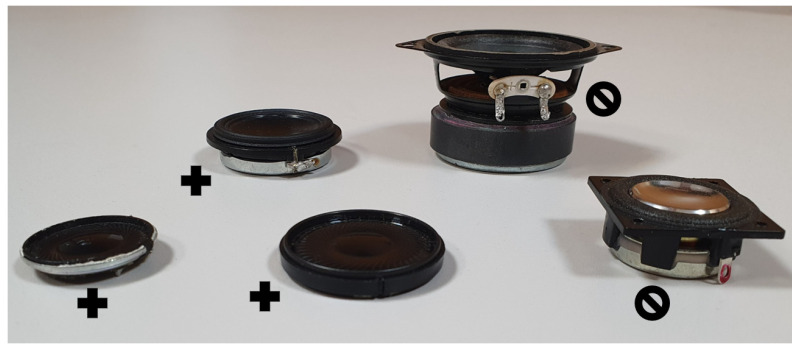
Comparison of the badly shaped transducers (crossed circles) and the design of the proper shape for the array loudspeakers (plus signs).

The selected designs for the miniature drivers used in the standard array design are shown in Figure 2; the plus signs indicate the correct shape for the transducer, and the crossed circles indicate the wrong type of driver.

## 3. Measurements of Electroacoustic Transducer Dispersions

**Figure 3:**
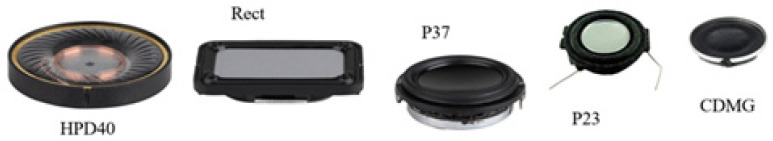
Photographs of the transducers selected for variability research.

In this section, the results of basic parameter measurements will be discussed. Some market models were selected to investigate the variability of commonly available transducers. They all comply with the requirements for the array loudspeaker matrix transducers explained in Section 2.2. Each transducer was purchased in 10 instances for the statistical investigation of parameter variability. Photographs of the selected transducers are shown in Figure 3.

The declared values of the selected transducers are gathered in Table 1.

To define the variability of the transducers investigated in this research, the coefficient of variation was calculated by the following Equation (4) [41]:

(4) $$\nu =\frac{\sigma }{\mu }*100\%$$

where the standard deviation is derived from the measurements and the average value is received (10 instances of each transducer measured). The higher the value, the greater the percentage of dispersion detected between the transducers in the trial. As standard deviation and statistical analyses were performed for each dataset (10 values), the normality test was performed using the Shapiro–Wilk method [42]. The tested data passed the normality test, so it is possible to use the standard deviation and define the confidence intervals with it. 

Different methods were used for the acoustic measurements. The driver sensitivity parameter was the logarithmic value expressed on the dB scale. Therefore, only the standard deviation parameter was used, and the dispersion (the difference between the maximum and minimum values in the trial) was measured.

### 3.1. Electric Measurements—Thiele–Small Parameters

**Figure 4:**
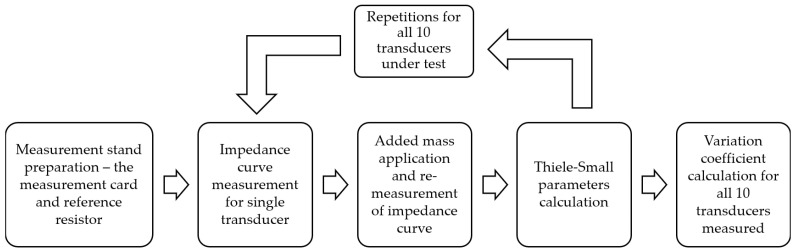
Block diagram of transducer dispersion measurement experiment—Thiele–Small parameters investigation.

The small-signal parameters of the selected drivers were measured using the standardized and well-known method of added mass. The Bruel & Kjaer PULSE analyzer was used with the reference resistor of 50 Ohm value and the required set of cables. The block diagram of the designed experiment is shown in Figure 4.

**Figure 5:**
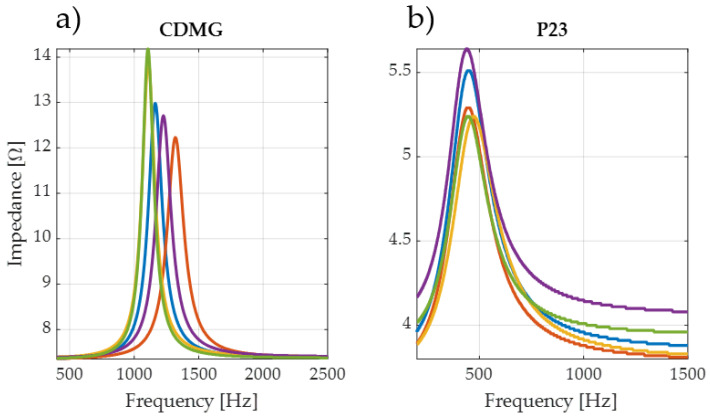
Comparison of example impedance curves: (**a**) high-dispersion transducer, (**b**) low-dispersion transducer. Different colors marks the different examples of transducers used in test.

The impedance curves were derived in two setups (with and without the added mass); this action was repeated for all 10 instances for each transducer, so 100 measurements were performed. Examples of measured impedance curves are shown in Figure 5.

Analysis of the impedance curve can provide significant information on the differences between the poor-repeatability transducers and the proper ones. The CDMG measurements show that resonant frequencies and maximum impedance parameters can differ significantly. The resonant frequencies may vary by as much as 350 Hz. The P23 is an example of proper transducer repeatability, where the resonant frequencies are close to each other and the impedance does not differ by more than 0.3 Ohm. 

**Figure 6:**
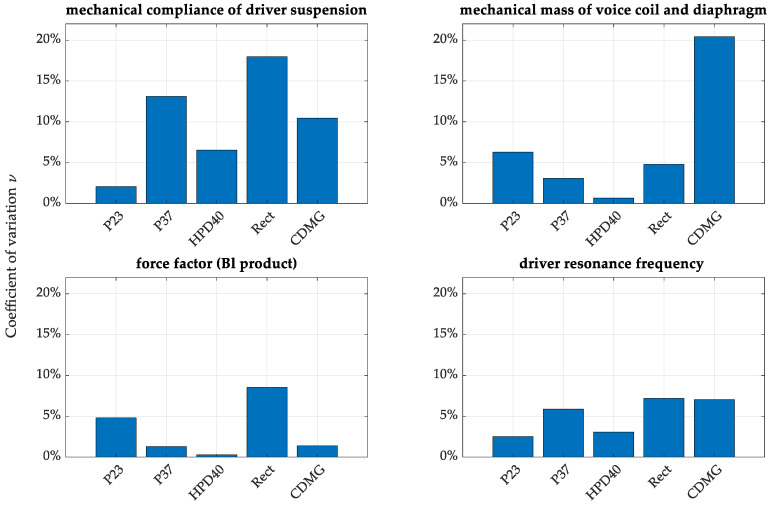
The coefficient of variation for Thiele–Small parameters for all measured transducers calculated based on 10 trials for each driver.

The essential aspect of this research is the comparison of the variation coefficients for all measured transducers, shown in Figure 6.

Regardless of the declared maximum dispersion from the nominal parameters, which was 10%, the research results proved that the variation may reach 20% for some significant T-S parameters. Of the analyzed transducers, the worst was the CDMG transducer, which showed almost 20% variation in *M_ms_* and 10% in *C_ms_*. The RECT transducer also showed bad ratings of 17% in *C_ms_* and 8% in BL factor. As the transducers tested differed in construction type (diaphragm material, basket type, shape, magnet type), they represent various approaches to transducer design—however, most of the transducers performed poorly in the experiment. Only a slightly better performance for the HPD40, the headphone driver, was noticed; it performed better than the other transducers tested. It is essential to note that the shift in the resonant frequency (typically around 5% in measured transducers) also affects the other parameters, which leads to error propagation. Based on the provided results, it is essential to note that all measured transducers should be individually measured for the T-S parameters and matched with similar ones to act as loudspeaker arrays or speaker sets.

### 3.2. Acoustic Measurements—Sensitivity

**Figure 7:**
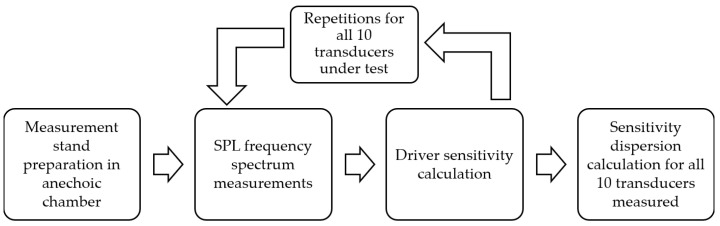
Block diagram of transducer dispersion measurement experiment—driver sensitivity investigation.

The last experimental study to be reported in the presented paper is the acoustic testing of the sensitivity parameter, the fundamental feature for the drivers, especially if they are supposed to be matched in a speaker set or an array. According to standard electroacoustic references, a set’s dispersion between the matched transducers should not exceed 0.5 dB [43,44]. To measure the dispersion between the analyzed instances of drivers, the anechoic chamber was measured at AGH University of Krakow under the procedure defined by the IEC standard [45]. The block diagram of the performed analysis is shown in Figure 7.

**Figure 8:**
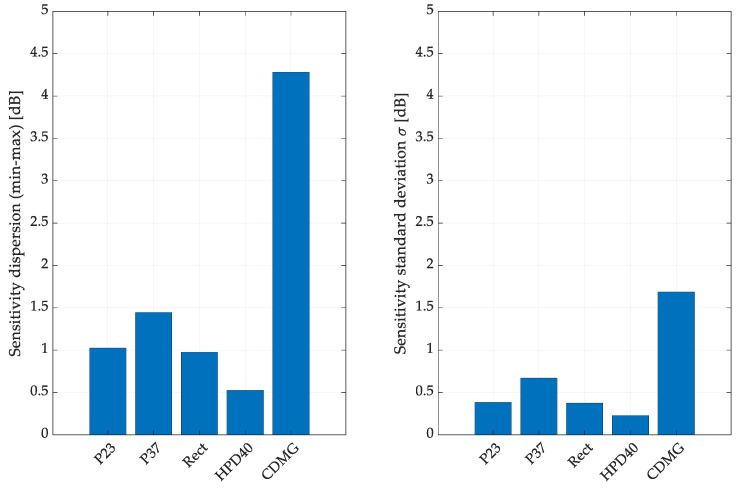
Driver sensitivity dispersion and standard deviation were calculated by measuring 10 individual instances for five transducers.

Only the absolute dispersion between the measured transducers and the standard deviation from the trial were analyzed to better investigate the needs of the logarithmic parameter dispersion analysis. The results are presented in Figure 8. 

The sensitivity measurements only partially confirmed the high dispersion detected in the electric parameter measurements. The second one performed significantly worse despite the similar variability in T-S parameters between the RECT and CDMG drivers. The standard deviation not being higher than 0.25 dB means that after the distance indicated by standard deviation is multiplied by 2 (so the 95% confidence interval), it is around 0.5 dB, which meets the reference requirement regarding the dispersion for array transducers. In the given research, only HDP40 met those conditions, and P23 and RECT transducers were close to meeting this criterion. The 95% confidence interval for P23, P37, and RECT was closer to 1 dB; for CDMG, it was 3 dB. Therefore, these dispersion thresholds were selected for the final numerical study.

## 4. Numerical Study of the Analysis of the Sensitivity Dispersion in the Performance of an Omnidirectional Sound Source

**Figure 9:**
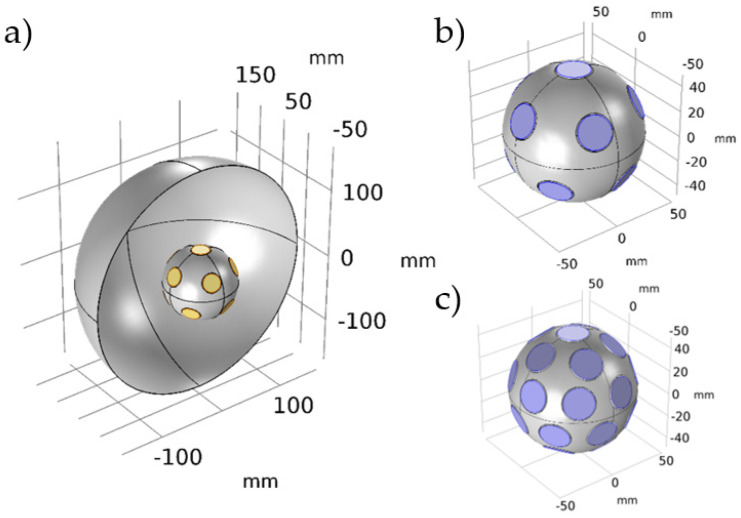
FEM model definition in COMSOL Multiphysics-(**a**) an overview of the environment and the tested sound source setups: (**b**) EQ12, (**c**) EQ36.

In this section, the overall influence of the transducer sensitivity influence was investigated when the omnidirectional sound source was considered. The initial assumption was that the significant dispersion between the transducers used in the matrix may influence the final source performance. To provide the data for analysis, we used Finite Element Method (FEM) modeling in COMSOL to obtain the source directivity data for the initial and modified matrix models. Example models of 12 (model EQ12) and 36 (model EQ36) omnidirectional source matrixes were prepared, where the transducers were placed in the sphere by using equal sphere partition algorithms [46]. Previous research first investigated the methodology [37,47]. The Pressure acoustic module in COMSOL [48] calculated the sound field around the simulated object. In these models, the enclosure surface was assumed to be perfectly rigid, while the transducers were represented as cylindrical disks positioned on the enclosure surface, with acoustic velocity conditions applied to this surface. In the basic model, all elementary sources had the same velocity applied, while in the ‘dispersed’ model, a different velocity was applied to each disk to simulate the applied dispersions of the transducers. Subsequently, these prepared models were situated within an air sphere of 1000 mm radius, with Perfectly Matched Layer (PML) conditions enforced along the boundaries. Then, sound pressure level (SPL) calculations were carried out on the sphere encompassing the 1000 mm distance, utilizing a 2-degree resolution for both azimuth and elevation angles. The model specifications for the chosen EQ partition models are shown in Figure 9.

The predominant technique used to assess the directivity of a sound source is as defined in the ISO 354 standard [49]. However, this method is commonly employed for conducting measurements in diffuse fields. Approaches aligned with established standards are utilized for analyzing commercial sound sources intended for on-site measurements. These methodologies incorporate various “averages” and “smoothing” processes that enhance the final performance metric. However, they can also inadvertently allow inferior sources to meet the requirements set by standardized tests. Limited techniques exist for evaluating the omnidirectional properties of a source based on fundamental statistical parameters. In the context of the present study, the standard deviation of the area-weighted levels (*σ_AWL_*) is adopted, consistent with the methodology outlined in [11,50], and is described by Equation (5):

(5) $$\sigma_{AWL}\left(f\right)=\sqrt{\frac{\sum_{m=0}^{M-1}\sum_{n=0}^{N-1}S_{m,n}{\left[L_{m,n}\left(f\right)-{\langle L_{m,n}\left(f\right)\rangle }_{S}\right]}^{2}}{\sum_{m=0}^{M-1}\sum_{n=0}^{N-1}S_{m,n}}},$$

where *M* and *N* represent the total number of measurements carried out in a three-dimensional space (which can include elevation and azimuthal angles), *L_m,n_ (f)* corresponds to the SPL at a particular point *m*, *n*, *Lm*, *n (f)* denotes the average SPL—whether measured or calculated—on a sphere of a specified radius, and *S_m,n_* indicates the segment of the area of the sphere corresponding to the point *m*, *n*. Consequently, *σ_AWL_* serves as a frequency-specific quantitative measure that reflects the uniformity of the source’s intensity in all directions. A higher value of *σ_AWL_* indicates that the sound source is not omnidirectional within the designated frequency range. Leishman et al. [8] have proposed that a *σ_AWL_(f)* value exceeding 1 dB should be considered as the threshold to determine the omnidirectional characteristics of the source.

**Figure 10:**
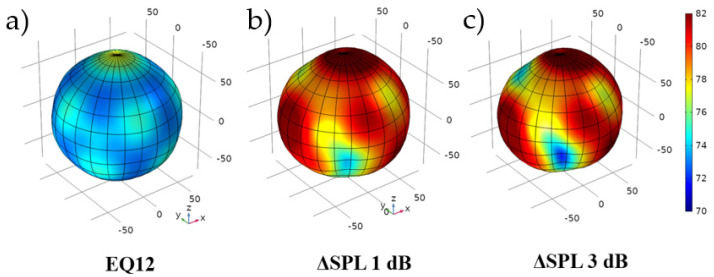
Sound source directivity plots for the EQ12 model and the frequency 4 kHz-(**a**) the reference model, (**b**) the model with sensitivity dispersion applied at the 1 dB level, (**c**) the model with sensitivity dispersion applied at the 3 dB level.

The following method was applied to investigate the influence of sensitivity dispersion in the study case. For each transducer, a different excitation level was used. Two levels of sensitivity mismatch were used: ΔSPL 1 dB and ΔSPL 3 dB. To simulate the mismatch in the numerical model, the levels for all 12 or 36 transducers were drawn from the normal distribution considering the two standard deviations borders of 1 dB or 3 dB, respectively, which means that 95% of the results were within the previously measured dispersions of sensitivity in Section 3. The example directivity plot, which visualizes the influence of dispersion between the transducer sensitivity for the EQ12 model, is shown in Figure 10.

**Figure 11:**
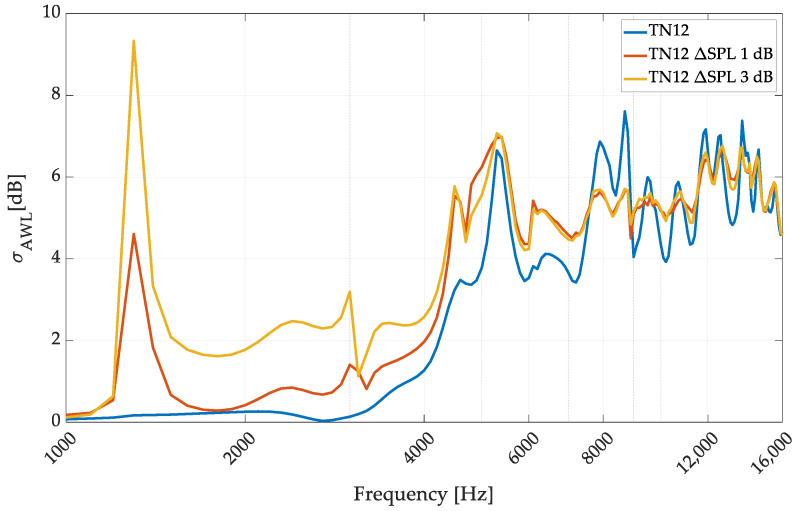
Omnidirectional performance evaluation of the EQ12 speaker array configuration with the applied transducer dispersion simulations.

To perform a strict assessment of the dispersion of the influence of transducers on the omnidirectional performance of the investigated array, *σ_AWL_* analysis was performed. The frequency range of 1000–16,000 Hz was analyzed, as, below 1000 Hz, we do not observe significant deviations from omnidirectionality, while 16,000 Hz is the upper limit that the omni-sources are used at so far [23]. The results of EQ12 modeling in the three investigated states are shown in Figure 11.

The simulation proved that applying the slight variation ΔSPL 1 dB significantly influenced the omnidirectional sound source performance. However, it does not significantly affect the omnidirectionality threshold considered by Leishman, but in the range of 2000–4000 Hz, the source provided a performance close to 1 dB for *σ_AWL_*, which was significantly worse and close to the utility border of the source. Using the ΔSPL 3 dB setup destroyed the omnidirectional performance of the source at middle frequencies. Of the five measured transducers, three could be qualified as ΔSPL 1 dB, and one as ΔSPL 3 dB (the remaining one was around ΔSPL 2 dB—not tested). This means that in a 12-transducer setup, if one of the measured transducers is used, then it will provide an unusable omnidirectional sound source. It is also essential to note that the high-frequency range of the transducer directivity test was not affected at all. Because of the high-order modes and, in general, high-directivity dispersion in this region, the influence of the sensitivity dispersion between the transducers can be neglected.

**Figure 12:**
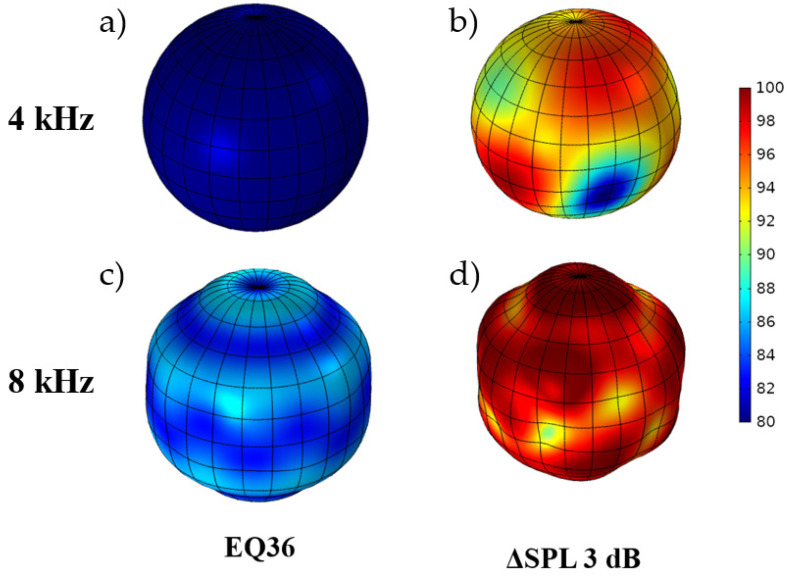
Sound source directivity plots for the EQ36 model: (**a**) no dispersion, 4 kHz; (**b**) ΔSPL 3 dB, 4 kHz; (**c**) no dispersion, 8 kHz; (**d**) ΔSPL 3 dB, 8 kHz.

The EQ36 configuration, i.e., the 36 transducers, was investigated in the following numerical study. Directivity plots for this setup are shown in Figure 12.

**Figure 13:**
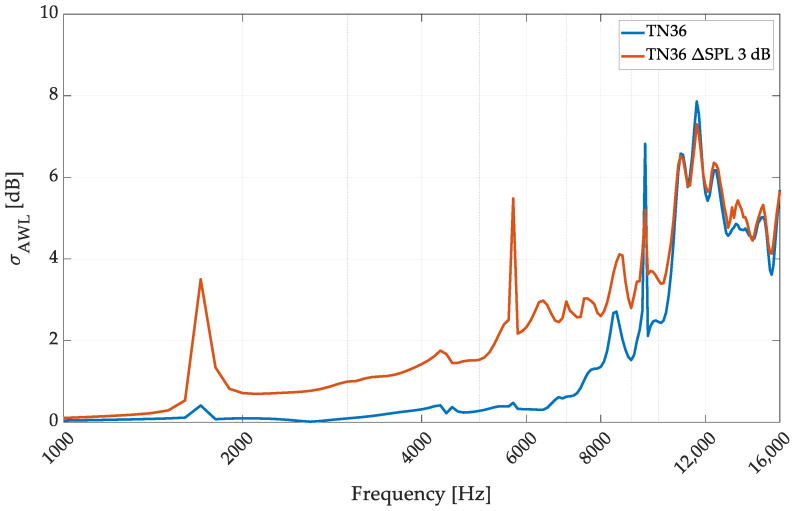
Omnidirectional performance evaluation of the EQ36 speaker array configuration with the applied transducer dispersion simulation.

In the EQ36 case, only the ΔSPL 3 dB case was studied, as it was used to confirm the previous finding, and the EQ36 configuration is less common in omnidirectional sound sources. The directivity plot analysis explains the phenomena generation of the higher dispersion of SPL on the sphere around the source while the sensitivity dispersion is applied. The analyzed case confirms that the mid-frequency range is the most affected, below the omnidirectionality cut-off frequency, around 8000 Hz in the EQ36 case and 4000 Hz in the EQ12 case. A detailed analysis of the directivity is shown in Figure 13.

Surprisingly, in the 2000–4000 Hz frequency range for the EQ36 setup, the ΔSPL 3 dB showed similar changes to ΔSPL 1 dB in the EQ12 case. The *σ_AWL_* did not grow to more than 1 dB, so the source maintained omnidirectionality, but above 4000 Hz, the influence of the transducer mismatch caused a significant deviation in omnidirectionality. The more negligible influence of the sensitivity mismatch in the EQ36 configuration can be explained by the phenomena that, in this case, the cross-interference between many transducers provides similar deviation to those caused by sensitivity mismatch; the mismatch application does not cause such significant changes in the source directivity.

## 5. Discussion

This research investigated the possible dispersions in driver parameters and their impact on loudspeaker arrays. It is essential to note that the dispersions measured at the T-S parameter measurement stage are not always directly connected with the sensitivity dispersion, which has the most significant impact on the acoustic performance of the array. Regardless of the high dispersions shown by four out of five tested models in the T-S parameter test, their sensitivity dispersions remained below 1 dB, which was an acceptable result for further array construction. This is essential, as T-S transducers are frequently randomly tested on production lines [51]. Sensitivity tests are rare, as they are challenging to conduct and require an anechoic environment, which is impossible to achieve on a mass production site. Therefore, it is insufficient to use only T-S parameter measurement in the transducer matching processes; sensitivity measurement is also needed. 

T-S dispersion may not be essential, but it is crucial to note that in the given research, the speakers assembled into the array in Section 4 shared the same volume of the enclosure. If the speaker were to be assembled into individual enclosures, in the situation common for most speaker sets for hi-fi or studio purposes, then the dispersion of T-S parameters could be crucial. The detected difference in parameters such as *C_ms_* around 15% will propagate this issue to the VAS parameter, which is crucial to the enclosure design [52,53]. Therefore, in the case of individual enclosures, the dispersion between the acoustic operation of the selected transducers may be even higher and should be tested in future research.

Based on the methodology provided in this article and the detected mismatches, it may be possible to develop models that compensate for the influence of the parameter dispersions in the individual drivers. However, a preliminary transducers check is needed when complete knowledge is required about the drivers to be used. Simple models with an output driving voltage limitation for each transducer could resolve the sensitivity dispersion reduction.

## 6. Conclusions

The presented research investigated the possible dispersion in electroacoustic transducer parameters, such as small-signal parameters and the driver’s sensitivity. A brief statistical analysis was performed by studying the 10 instances of five different transducer models, which provided information about the possible dispersion of these parameters in empirical measurements. Also, a detailed numerical study of the dispersion influence on sound source directivity was conducted to show the phenomenon’s influence on omnidirectional sound sources. Performing the research described in the current paper allowed us to derive several conclusions regarding the loudspeaker selection process in array construction. The typical variation in the T-S parameters in electroacoustic transducers does not exceed 5%; however, it may reach 15% or 20% for selected parameters. The dispersion is not associated with the type of transducer or other detected dispersions. However, if the transducers are placed in individual enclosures, then the dispersion of the T-S parameters may be significantly more critical.

It was detected that variation in T-S parameters does not correlate directly with the dispersions of the acoustic sensitivity parameter. While 15–20% variation in T-S parameters was selected for some transducers, the sensitivity dispersion remained below 1 dB dispersion in absolute values and 0.4 dB in the standard deviation of the trial. However, it is essential to note that the sensitivity tests were performed in an infinite open baffle, while the T-S parameters may also affect the enclosure design. Further study is required in this case.

Numerical directivity tests proved that the sensitivity dispersion at the level of 1 dB does not significantly affect the performance of the omnidirectional sound sources or any other multitransducer matrix. At the same time, the higher mismatch provides a significant decrease in the measured configuration performance. It is safe to proceed with transducers that do not provide dispersions higher than 1 dB, but this should be preliminarily tested on the given transducer samples before the array is constructed, to avoid performance problems.

Research has shown that parameter mismatches caused by poor transducer manufacturing tolerance and the dispersion of material parameters may significantly affect the electroacoustic design process of enclosures or the final sound source construction. Therefore, it is essential to performing transducer matching or randomized acoustic tests for selected measured transducers to reveal possible dispersions before matrix construction. Future work on this higher sensitivity could cover the further investigation of selected T-S parameter mismatches in the enclosure design process, as selected dispersions of 15–20% may result in significantly higher-sensitivity dispersions if the transducers are used in enclosures, not in infinite baffles.

## Figures and Tables

**Table 1:** Essential parameters of drivers used in the research.

Sample Name	Manufacturer Code	*f_s_* [Hz]	*M_ms_* [g]	*C_ms_* [s^2^/kg]	BL [Tm]	Sensitivity [dB]
HPD40	HPD-50N25PR00-32	69.5	0.7	7.5	2.77	98.1
P37	PMT-37N28AL01-04	167.8	1.2	0.4	2.97	89.8
P23	PMT-30N18AL04-04	249.7	0.13	0.98	0.51	78.5
CDMG	CDMG16008-03	600.0	0.006	0.79	0.31	69.2
Rect	PMT-2040N1625AL01-04	320.2	0.4	0.6	1.23	83.9

## Data Availability

Data are available on request directly from the author.
